# Promoting Teachers’ Social and Emotional Competence in Light of the Close Connection between Professional Role and Personal Characteristics: Preliminary Evidence of the Efficacy of the “ME4YOU” Training Program

**DOI:** 10.3390/ijerph21040511

**Published:** 2024-04-21

**Authors:** Enrica Ciucci, Carolina Facci, Daniela Carpenzano, Matilde Sanesi, MariaGiulia Taddei, Lucrezia Tomberli, Giovanna Tambasco, Andrea Baroncelli

**Affiliations:** 1 Department of Education, Languages, Interculture, Literatures and Psychology, University of Florence, 50121 Florence, Italy; enrica.ciucci@unifi.it (E.C.); carolina.facci@unifi.it (C.F.); daniela.carpenzano@unifi.it (D.C.); lucrezia.tomberli@unifi.it (L.T.); 2 Independent Researcher, 51100 Pistoia, Italy; matilde.sanesi@libero.it (M.S.); taddeimariagiulia@gmail.com (M.T.); 3 Non-Profit Organization EbiCo, University of Florence, 50123 Florence, Italy; tambasco.giovanna@yahoo.it; 4 Department of Philosophy, Social Sciences and Education, University of Perugia, 06123 Perugia, Italy

**Keywords:** social and emotional competence, well-being, teachers, school system, training program

## Abstract

Within the field of research on the promotion of teachers’ social and emotional competence, the present paper illustrates preliminary evidence of the efficacy of a new training program named “ME4YOU” aimed at supporting teachers’ self-reflexive competences to deal with the emotional and relational dimensions of teaching, with constant and continuous attention towards underlining the close connection between the way teachers perform as professionals at work and the way they function as individuals in their personal life. A total of 109 teachers from kindergarten to primary school took part in the experimental group, while 67 teachers constituted the control group; the two groups were compared using a pre-test/post-test approach with regard to some self-reported variables related to professional and personal aspects. Teachers in the experimental group exhibited increased levels of professional self-efficacy and self-efficacy as emotional socializers toward students’ emotions; moreover—although with a more limited impact—they reported benefits with regard to their personal life (i.e., reduced denial of own emotions and improved authenticity). The findings are discussed highlighting that health promotion among teachers is both of value in itself and an investment that can generate health in the whole school system.

## 1. Introduction

The promotion of teachers’ well-being and improvements in their social and emotional competence are considered key aspects that influence the development of their students and promote health and well-being in the whole school system [1,2,3,4]. According to the Prosocial Classroom Model by Jennings and Greenberg [3], both personal and professional teachers’ characteristics are activated when they cope with the multiple job demands that arise from the school context. There is no doubt that a teacher must possess solid professional preparation and specialized knowledge for effective teaching, starting an intervention program with students, or relating to other adults at school; it is equally important to recognize that these professional skills go along with a teacher’s broader characteristics as a person, which include a unique set of emotional experiences, beliefs, strengths, and fragilities. For instance, with reference to the social and emotional domain, teachers constantly refer to their personal emotional competence in order to recognize, express, and regulate both their own and others’ emotions at work, therefore needing to develop awareness of clear representations of the boundaries between their personal life and professional role [5]. The present paper illustrates the initial evidence of the efficacy of a psychological training program—named “ME4YOU [ME for YOU]: Take care of ME to take care of YOU. Teacher’s well-being for the promotion of well-being at school”—aimed at supporting teachers’ well-being and developing self-reflexive competences to deal with the emotional and relational dimensions of teaching, with constant and continuous attention towards underlining the close connection between the way teachers perform as professionals at work and the way they function as individuals in their personal life.

A well-established field of research has shown that teaching is one of the most stressful occupations [6,7,8]. Involving interactions and relationships between people, teaching inevitably activates an important emotional dimension: school settings can be considered complex emotional arenas where teachers encounter emotional challenges with students’, colleagues’, deans’, and parents’ demands, and where they are called to manage both their own and others’ emotions and behaviors [2,3,9]. In the process, teachers not only have to apply specific didactic or professional techniques, but they have also to deal with the emotional resonances that inevitably arise. Moreover, many teachers have a strong emotional investment in their work in terms of values, ethics and moral objectives: teaching may become one of the main sources for their self-esteem and fulfillment, and sometimes, it can lead to vulnerability to stress and burnout in the presence of a maladaptive fusion between the sense of professional identity and the sense of personal identity [5,9]. Further, teachers are subject to expectations from others regarding their emotional functioning. The construct of emotional labor in the teaching profession highlights that teachers experience continuous emotional strain to generate, regulate, inhibit, and express their emotions in accordance with the normative beliefs and expectations that people have about teaching, and this has a strong influence on their job satisfaction and their physical health and well-being, as well as on the adjustment of their students (i.e., in terms of students’ social adaptation, emotional competence, and academic outcomes) [10,11]. Consequently, teachers have to develop a balance between responding to the expectations of others and acting in a way that respects their own emotional states and beliefs; this process is strictly related to the construct of authenticity, which refers to the congruence between one’s own internal states, awareness of one’s own true Self, and the expression of such states [12]. Extant research has shown that experiencing inner states of authenticity at work promotes more awareness, self-determination, and proactivity toward own profession [12,13]. Consequently, supporting teachers in (a) positively managing relationships with others by considering the emotional resonances that are activated, (b) gaining an awareness of their emotional investment in the profession and its effect on their personal functioning, and (c) handling their emotional labor in the direction of greater authenticity should be a significant practice during their in-service training. The ME4YOU training program operates in this direction by promoting teachers’ reflexivity, which refers to the critical monitoring of themselves in interactions with others during daily educational practices—both while they are underway and afterwards—in order to gain greater awareness of their own functioning that is used to rethink and improve own educational actions [14,15,16,17]. As suggested by the literature [14,17], teachers are often encouraged to actively reflect on technical teaching actions, e.g., with reference to what has favored or hindered a specific performance in students; however, reflection does not guarantee a real change in the beliefs or in the practices. A deeper elaboration is supported by the process of reflexivity: by sustaining in teachers the development of awareness about their broader social and emotional characteristics, reflexivity emerges at the same time as a source that informs the Self, and as a basis for a critical and transformative rethinking of one’s way of being and acting as a professional. In their attempt to clarify the difference between reflection and reflexivity, Feucht and colleagues emphasize that “*Reflection becomes reflexivity when informed and intentional internal dialogue leads to changes in educational practices, expectations, and beliefs*” [14], p. 234. Reflexivity therefore requires a high degree of intentionality and can be actively promoted in teachers through specific training activities.

How to deal with reflexivity concerning emotions and relationships in teaching necessarily calls into question the construct of teachers’ social and emotional competence. Social and emotional competence is an umbrella term that reflects self-efficacy in dealing with emotion-eliciting social transaction [18]. Specifically, according to the extensive research of the Collaborative for Academic, Social, and Emotional Learning (CASEL), it refers to five interrelated competencies: to understand and manage own emotions (self-awareness and self-management), to understand others’ emotions and show empathy for them (social awareness), to develop and maintain positive relationships (relationship management), and to make responsible decisions (responsible decision making) [19,20]. The approach promoted by the CASEL has initially focused on students and has resulted in several evidence-based training programs that have shown a positive impact on students’ mental health and well-being [21,22]. However, it has immediately become clear that teachers’ well-being and personal competences play a central role in this process, so that preconditions for teachers to effectively implement these programs are to take care of their own social and emotional competences [3,4,23,24]. In fact, teachers with high levels of social and emotional competence show high self-awareness and are able to use pleasant emotions (i.e., joy and enthusiasm) to motivate themselves [3,4]. Moreover, teachers high in social and emotional competence have high social awareness: they recognize and understand the emotions of others and are aware that others may have different perspectives than they have, taking this into account in relationships with students, parents, and colleagues; they also know how to manage their emotions and behaviors to motivate others [3,4].

Deepening the theme of own and others’ emotions in educational and scholastic contexts, a recent field of research has explored the construct of meta-emotion philosophy in the student–teacher relationship [25,26]. Specifically, meta-emotion philosophy theorizes an emotional bond between caregivers and children: caregivers’ beliefs and feelings about emotions are expressed into behaviors toward own emotions as well as toward children’s emotions [27]. Originally explored within the parent–child relationship, this field of research has shown that adults who deny their emotional experience may convey this disposition through dismissing style and critical responses to children’s emotions, through modeling avoidance, and through teaching the value of emotion suppression; on the other hand, adults who openly accept their emotional experience are likely to encourage the same model of emotions in children [27,28,29]. This construct has also been recently investigated in the student–teacher relationship, highlighting that crèche educators and teachers (from kindergarten to middle school) with high self-efficacy in relation to their own emotions in their general life are more prone to taking care of students’ emotions (i.e., both the pleasant and the unpleasant ones) and have higher self-efficacy with reference to their role of emotional socializers toward students (i.e., they highly value their role in students’ social and emotional development and feel confident to successfully behave accordingly); on the contrary, teachers with higher levels of rejection of their own emotions feel less confident in their role as emotional socializers [25,26]. To summarize, teachers’ social and emotional competence seems to be the driving force of an adaptive process which, in cascade, affects the way through which they recognize students’ emotions and needs, develop warm and supportive relationships with them, effectively implement the most appropriate social and emotional interventions and manage their classrooms effectively, and promote well-being and health in the whole school context [3]. Thus, it appears crucial to support teachers in the caring of their own social and emotional competence and well-being.

### 1.1. The ME4YOU Training Program

On the basis of the above-reported literature, it appears clear that investing in the caring of teachers’ social and emotional competence and well-being represents both a caring practice on the part of a school organization that aims to promote health among its professionals, and an investment to generate health in the whole system. Even if it is out of the aim of the present paper to review all the training programs devoted to take care of teachers’ social and emotional competence, it is to highlight that in the past few years several interventions have been implemented and evaluated. For instance, two examples of training programs inspired by the mindfulness approach that proved to impact on teachers’ social and emotional competence are the CARE (Cultivating Awareness and Resilience in Education) [30], which helps to face stressors at work and to build awareness about own emotional functioning, and the SMART-in-Education (Stress Management and Resiliency Training) [31], which is focused on self-care practices to help teachers with daily stressors and to promote emotional control and resilience. Another recent promising training program is the PROMEHS (PROmoting MEntal Health at Schools) [23], designed to address students’ and teachers’ mental health through training workshops and handbooks; specifically, training activities for teachers are focused on how promote mental health at school, with specific attention also on how to take care of one’s own mental health and resilience. In addition to having a positive impact on students, recent evidence highlights that PROMEHS has also an impact on teachers’ mental health outcomes, such as social and emotional learning, resilience, and self-efficacy [23]. To further contribute to this field of research and intervention, we developed the ME4YOU training program with the general aim to sustain reflexivity in teachers in (a) positively managing relationships with others by taking into account the emotional resonances that are activated, (b) gaining an awareness of their emotional investment in the profession and its effect on their personal functioning, and (c) handling their emotional labor in the direction of greater authenticity. We believe that this program can offer a training experience in which teachers can gain more mature awareness of their social and emotional functioning and build bridges between theory and practice, such that it can affect their own beliefs and emotional experiences; in this regard, the specific aim of the ME4YOU training program is to modify teachers’ self-efficacy with regard to professional and personal aspects (i.e., professional self-efficacy, self-efficacy as emotional socializers, and self-efficacy toward one’s own emotions) as well as the way they approach their own emotions (i.e., in terms of the denial of emotions and authenticity).

The ME4YOU program is an in-service professional training proposal developed in Italy for teachers coming from kindergarten, primary school, and middle school (i.e., in Italy, these three school levels are usually part of the same comprehensive scholastic institutions). It is made up of eight meetings (2.5 h each) over approximately 3 months, and it is administered by a trained psychologist—with experience in the field of developmental psychology and school settings—to the same group of teachers (of approximately 15–20 participants). The program is evaluated using a quasi-experimental approach involving an experimental group (i.e., teachers that register voluntarily, without economic incentives, and attend at least six of the eight scheduled meetings) and a control group (i.e., teachers who do not attend the program and voluntarily served in the control group after being invited by the school deans or their collaborators). The experiential activities focus on challenges or relational situations that refer to constructs that are central to social and emotional dynamics at school with students, students’ families, deans, and colleagues (e.g., locus of control, self-efficacy, communication strategies, stress and coping). In each meeting, after an initial part aimed at bringing attention to the present moment through exercises of concentration and breathing, the reference construct of the day is briefly introduced from a theoretical point of view and discussed with teachers, and then an experiential activity is presented (i.e., participants get involved with the whole group in solving a problem, facing a challenge, or accomplishing a task). Experiential activities always start from situations related to real school life (e.g., a communication problem with a colleague or a student) but require the activation of dynamics that go beyond the school dimension (e.g., actively experimenting with different communication techniques, evaluating the impact they have in building a relationship, reflecting on those that they use most in the various contexts of their life and what could be the concrete changes brought by a use of different techniques); in this way, they stimulate reflexive insight consisting of both understanding the reciprocal interconnections between professional role and personal characteristics and developing new beliefs and approaches to own functioning as a professional and a person. We highlight that an important aspect of the ME4YOU training program is to promote reflexivity through group interactions. In this regard, a fundamental premise for all activities is to build a climate of trust, respect, and confidentiality among participants, such that everyone feels free to participate according to her/his own style and with the guarantee of being respected. This approach also allows teachers to share with peers their school experiences and to experience less loneliness, developing the awareness that often they face very similar challenges and stressors. If necessary, participants can also request a moment of individual discussion with the trainer, to delve deeper into dynamics or salient points that emerged during the meetings or as the result of personal reflection.

### 1.2. The Present Study

The central aim of the present study was to provide initial evidence of efficacy of the ME4YOU training program with reference to five psychological constructs that refer to teachers’ social and emotional competence and well-being. First, we focused on three different facets of self-efficacy: two of them mainly capture work-related aspects (i.e., professional self-efficacy and self-efficacy as emotional socializers), while the third pertains general aspects of own emotional life (i.e., self-efficacy toward own emotions). Self-efficacy refers to the subjective perceptions and beliefs of one’s own capability to perform specific activities or gain desired goals, and it has a domain-specific nature [32,33]. Despite self-efficacy being an individual characteristic with a certain degree of stability, it is not fixed and immutable: its development depends on both intrapersonal and interpersonal factors (i.e., including feedback from others), thus resulting dynamic as it can change due to novel information and experiences [32,33]. In other words, self-efficacy can be improved, and we predicted that activities such as those of the ME4YOU—which both constantly stimulate participants to actively get involved in an experiential way and require the training group to exercise continuous realistic and caring feedback—can constitute fertile ground for its development. Specifically, we hypothesized that the contents of the experiential activities had a positive impact on both perceptions and beliefs concerning one’s own ability to successfully cope with general tasks and challenges related to one’s own professional role (i.e., professional self-efficacy [34]), with particular attention on a deeper understanding of the impact on students’ developmental trajectories (i.e., self-efficacy as emotional socializer [25]); further, we hypothesized that discussing and expressing oneself openly, while respecting own emotional and relational style, provide participants with greater security in dealing with their own emotions (i.e., emotional self-efficacy toward own emotions [25]). Moreover, we focused on further two constructs related to the functioning in the general life: denial of own emotions and authenticity. Specifically, we hypothesized that the nature of the experiential activities proposed puts participants in contact with the entire range of emotions—from pleasant to unpleasant ones—providing skills to accept and manage them, and therefore reducing the rejecting or not accepting one’s own emotional experience (i.e., denial of own emotions [25]). At the same time, we hypothesized that the ME4YOU provides participants with emotional training in which, by expressing themselves freely and without fear of being judged, they can contact the most authentic parts of their Self, thus strengthening personal and professional posture to be able to stay in conscious contact with own internal states and knowing how to express them in an adaptive way (i.e., authenticity [12]).

## 2. Materials and Methods

### 2.1. Participants and Procedure

In the present study, we considered an experimental group of 109 teachers who took part in 7 different groups of the ME4YOU training program (103 females; M professional experience = 15.47 years, SD = 9.27 years; 42 from a kindergarten, 36 from a primary school, 31 from a middle school) and 67 teachers who did not take part in the ME4YOU program and constituted the control group (62 females; M professional experience = 15.25 years, SD = 8.84 years; 27 from a kindergarten, 31 from a primary school, 9 from a middle school). Three different phases were involved in the evaluation of the efficacy of the training program: in the pre-test phase, teachers of both experimental group and control group completed the informed consent form (i.e., for the experimental group only, the consent also covered in detail all aspects related to the participation in the training program) and the psychometric questionnaires; the second phase involved the realization of the training program for teachers in the experimental group, and a theoretical meeting on teachers’ social and emotional competence and well-being for those in the control group; in the post-test phase, the same psychometric questionnaires were completed again by teachers in the experimental group and in the control group after about 15–20 days after the end of the training activities. In each phase, teachers completed questionnaires using an online form that was sent to their email address by the trainer; to ensure confidentiality, participants were provided with an alphanumerical code to use in the pre-test and post-test assessment process. The main results that emerged were shared with schools during seminars focused on the theme of well-being at school.

### 2.2. Measures

#### 2.2.1. Teachers’ Professional Self-Efficacy

This domain of self-efficacy was investigated using the 6-item self-report questionnaire by Caprara [35]: teachers had to indicate their agreement with each item using a 5-point Likert-type scale, from “not at all” (1) to “very much” (5). Items investigated different work-related tasks and challenges that teachers daily face (e.g., “I am able to integrate effectively with colleagues”; “I am able to successfully solve the problems posed by my work, even the most demanding ones”). A mean score of all items was calculated. Cronbach’s alpha in the present sample was 0.81 at the pre-test and 0.73 at the post-test.

#### 2.2.2. Teachers’ Self-Efficacy as Emotional Socializer toward Students

We adopted a specific 6-item subscale of the Crèche Educator Emotional Style Questionnaire—version for teachers (CEESQ) [25] to assess teachers’ self-perception related to their professional role and capabilities in the socialization of students’ emotions (e.g., “I easily recognize the emotions that a student is experiencing”, “I feel able to help students cope with their fears and their anger”). A 5-point Likert-type scale (from 1 = “not true” to 5 = “completely true”) was used. A mean score of all items was calculated. Cronbach’s alpha in the present sample was 0.83 at the pre-test and 0.81 at the post-test.

#### 2.2.3. Teachers’ Emotional Self-Efficacy concerning Their Own Emotions

Teachers’ emotional self-efficacy concerning recognition, expression, and regulation of one’s own emotions in general life (e.g., “I can easily identify the reasons for my emotions”; “I am able to manage emotions that are too intense”) was investigated with a 10-item dimension from the above-presented CEESQ. Once again, a mean score of all items was calculated. Cronbach’s alpha in the present sample was 0.83 at the pre-test and 0.86 at the post-test.

#### 2.2.4. Denial of Own Emotion

Teachers’ level of denial of emotions in general life was once again assessed with a 5-item dimension from the CEESQ (e.g., “I don’t like the emotions I experience”; “I perceive my negative emotions as something to defend myself against”). A mean score of all items was calculated. Cronbach’s alpha in the present sample was 0.41 at the pre-test and 0.49 at the post-test.

#### 2.2.5. Authenticity

Teachers’ perception of authenticity in their life was assessed using the Italian version of the Authenticity Scale [36,37]. It is a 12-item self-report questionnaire to assess context-free perception of self-alienation (reversed; e.g., “I feel as if I don’t know myself very well”), authentic living (e.g., “I live in accordance with my values and beliefs”), and accepting external influence (reversed; e.g., “I am strongly influenced by the opinions of others”). Teachers expressed their agreement with each item using a 7-point Likert scale from 1 (“does not describe me at all”) to 7 (“describes me very well”). We calculated the mean of the 12 items. Cronbach’s alpha in the present sample was 0.81 at both the pre-test and the post-test.

### 2.3. Data Analyses

First, we inspected the distributions of the study variables by calculating skewness and kurtosis values. Since one of the characteristics of the present quasi-experimental approach was that it was not possible to randomly attribute the participants to the experimental group or to the control group, we first checked for statistical differences in the mean values at the pre-test with a series of analyses of variance (ANOVAs). After that, we investigated the impact of the training program on the study variables through a series of repeated-measures analysis of variance (ANOVA) with time (pre-test or post-test) as a within-subject factor and the group (experimental or control) as a between-subject factor. To conclude, we checked again for statistical differences in the mean values at the post-test with an additional series of analyses of variance (ANOVAs).

## 3. Results

All indices of skewness and kurtosis at both pre-test and post-test were in the expected range [−1.00;+1.00] in both the experimental group and the control group.

**Figure 1:**
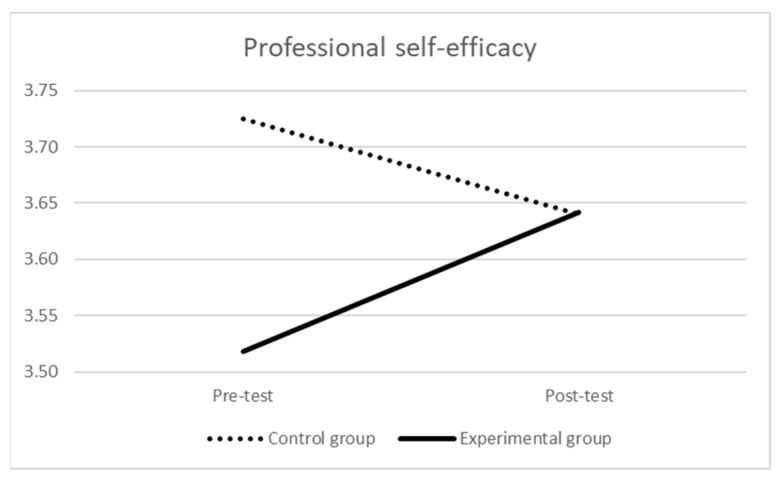
Professional self-efficacy over time by group.

As for professional self-efficacy (see Table 1 and Figure 1) at the pre-test, the mean score of the experimental group was significantly lower than the one of the control group (F (1,167) = 5.864, *p* = 0.017, η_p_^2^ = 0.03). The repeated-measures ANOVA indicated that the effect of time was not statistically significant (Wilks’ λ = 0.998, F (1,166) = 0.338, *p* = 0.562, η_p_^2^ = 0.002); nevertheless, a significant effect of the time × group interaction emerged (Wilks’ λ = 0.946, F (1,166) = 9.459, *p* = 0.002, η_p_^2^ = 0.05): there was a significant difference over time (i.e., increasing) for the experimental group (Wilks’ λ = 0.929, F (1,104) = 7.922, *p* = 0.006, η_p_^2^ = 0.07) and a near-significant difference over time (i.e., decreasing) for the control group (Wilks’ λ = 0.952, F (1,62) = 3.152, *p* = 0.081, η_p_^2^ = 0.05). At the post-test, the difference in the mean scores of the two groups was no longer statistically significant (F (1,167) = 0.001, *p* = 0.985, η_p_^2^ = 0.001).

**Figure 2:**
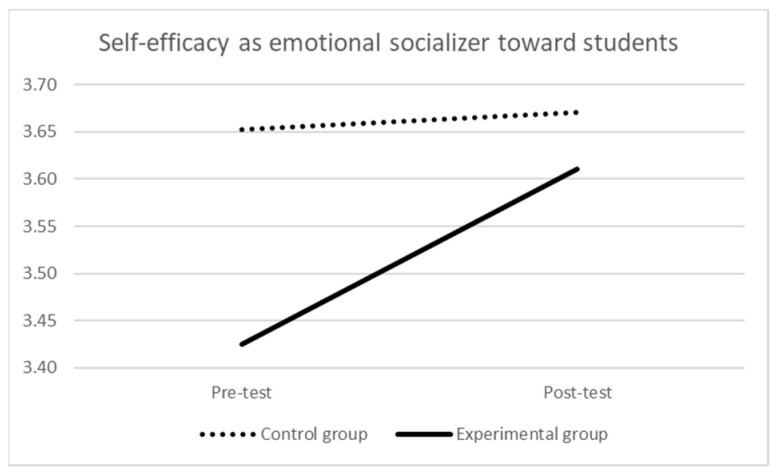
Self-efficacy as emotional socializer toward students’ emotions over time by group.

Considering self-efficacy as emotional socializer toward students’ emotions (see Table 2 and Figure 2), at the pre-test, the mean score of the experimental group was significantly lower than the one of the control group (F (1,175) = 8.388, *p* = 0.004, η_p_^2^ = 0.05). The repeated-measures ANOVA indicated a significant effect of time (Wilks’ λ = 0.937, F (1,174) = 11.717, *p* < 0.001, η_p_^2^ = 0.06), which was qualified by a significant time × group interaction (Wilks’ λ = 0.956, F (1,174) = 7.971, *p* = 0.005, η_p_^2^ = 0.04): there was a significant difference over time (i.e., increasing) for the experimental group (Wilks’ λ = 0.825, F (1,108) = 22.848, *p* < 0.001, η_p_^2^ = 0.18), while there were no differences for the control group (Wilks’ λ = 0.997, F (1,66) = 0.181, *p* = 0.672, η_p_^2^ = 0.003). At the post-test, the difference in the mean scores of the two groups was no longer statistically significant (F (1,175) = 0.632, *p* = 0.428, η_p_^2^ = 0.004).

**Figure 3:**
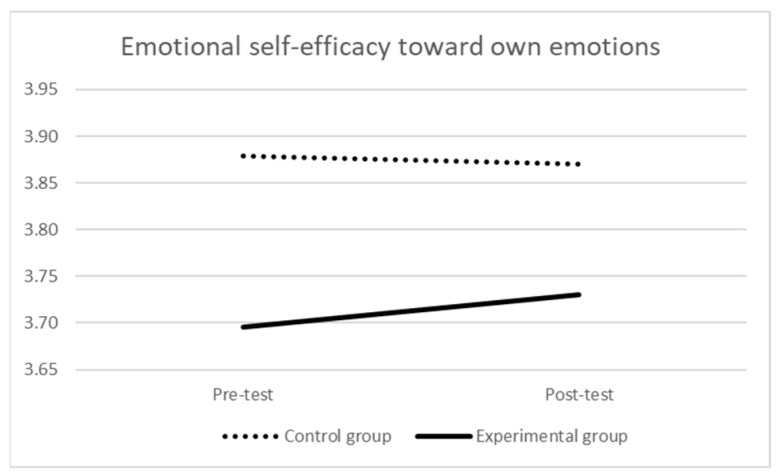
Self-efficacy toward own emotions over time by group.

Focusing on emotional self-efficacy toward own emotions (see Table 3 and Figure 3), at the pre-test, the mean score of the experimental group was significantly lower than the one of the control group (F (1,173) = 6.219, *p* = 0.014, η_p_^2^ = 0.04). The repeated-measures ANOVA indicated neither a significant effect of time (Wilks’ λ = 0.999, F (1,172) = 0.189, *p* = 0.664, η_p_^2^ = 0.001) nor a significant time × group interaction (Wilks’ λ = 0.997, F (1,172) = 0.548, *p* = 0.460, η_p_^2^ = 0.003). At the post-test, there still was a near-significant difference in the mean scores of the two groups (F (1,173) = 3.110, *p* = 0.080, η_p_^2^ = 0.018).

**Figure 4:**
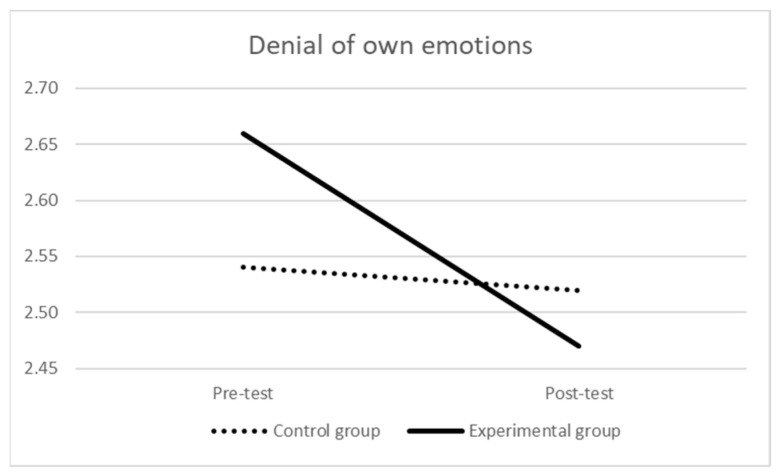
Denial of own emotions over time by group.

As for the denial of own emotions (see Table 4 and Figure 4), at the pre-test, the mean score of the experimental group was not significantly different than the one of the control group (F (1,171) = 1.573, *p* = 0.211, η_p_^2^ = 0.01). The repeated-measures ANOVA indicated a significant effect of time (Wilks’ λ = 0.969, F (1,170) = 5.458, *p* = 0.021, η_p_^2^ = 0.03) that was qualified by a near-significant effect of time × group interaction (Wilks’ λ = 0979, F (1,170) = 3.695, *p* = 0.056, η_p_^2^ = 0.02): there was a significant difference over time (i.e., decreasing) for the experimental group (Wilks’ λ = 0.901, F (1,106) = 11.665, *p* < 0.001, η_p_^2^ = 0.10), while the difference over time for the control group was not significant (Wilks’ λ = 0.999, F (1,64) = 0.072, *p* = 0.789, η_p_^2^ = 0.001). At the post-test, the difference in the mean scores of the two groups was still not statistically significant (F (1,171) = 0.361, *p* = 0.549, η_p_^2^ = 0.002).

**Figure 5:**
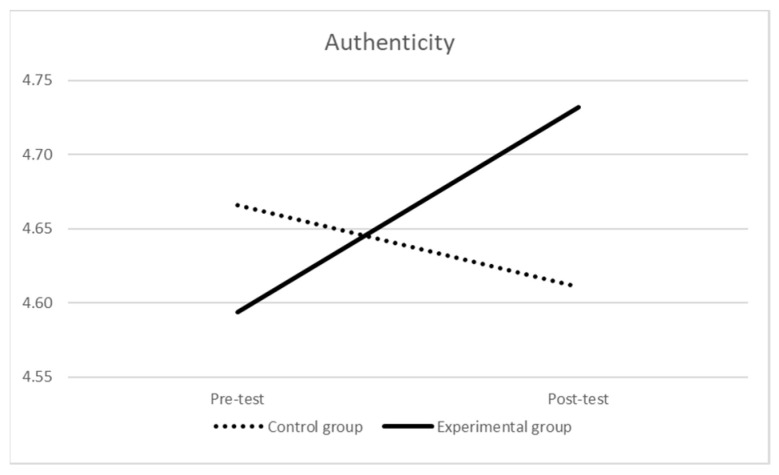
Authenticity over time by group.

Finally, considering authenticity (see Table 5 and Figure 5), at the pre-test, the mean score of the experimental group was not significantly different than the one of the control group (F (1,168) = 0.292, *p* = 0.590, η_p_^2^ = 0.002). The repeated-measures ANOVA indicated that the effect of time was not significant (Wilks’ λ = 0.996, F (1,167) = 0.652, *p* = 0.421, η_p_^2^ = 0.004); nevertheless, there was a near-significant effect of time × group interaction (Wilks’ λ = 0.979, F (1,167) = 3.610, *p* = 0.059, η_p_^2^ = 0.02): there was a significant difference over time (i.e., an increasing) for the experimental group (Wilks’ λ = 0.958, F (1,104) = 4.563, *p* = 0.035, η_p_^2^ = 0.04), while the difference over time for the control group was not significant (Wilks’ λ = 0.992, F (1,63) = 0.534, *p* = 0.468, η_p_^2^ = 0.01). At the post-test, the difference in the mean scores of the two groups was still not statistically significant (F (1,168) = 0.809, *p* = 0.370, η_p_^2^ = 0.01).

## 4. Discussion

The “ME4YOU: Take care of ME to take care of YOU. Teacher’s well-being for the promotion of well-being at school” training program was developed with the general aim to take care of the social and emotional competence and well-being of specific professionals: teachers. Specifically, the training program was expected to modify teachers’ self-efficacy with regard to both professional and personal aspects (i.e., professional self-efficacy, self-efficacy as emotional socializers, and self-efficacy toward own emotions) as well as the way they approach their own emotions (i.e., in terms of denial of emotions and authenticity). Starting from the well-established evidence that healthy and socio-emotionally competent teachers are the driving force of health processes that reach the entire school system (including students) [1,2,3,4], the ME4YOU program takes into account the emotional complexity that teachers daily deal with, and aims at supporting their reflexivity in (a) positively managing relationships with others and the related emotional resonances that are activated, (b) gaining an awareness of their emotional investment in the profession and its effect on their personal functioning, and (c) handling their emotional labor in the direction of greater authenticity. The consequentiality suggested by the subtitle (i.e., first “take care of ME”, and then “take care of YOU”) refers to the assumption according to which the preconditions for teachers to effectively manage emotions and relationships at work are to take care first of their own social and emotional competences [3,4,23,24]. The main aim of the present study was to provide initial evidence of the efficacy of the ME4YOU program by investigating whether an experimental group of teachers that undertook the program—compared with a control group—showed an improvement in some indicators connected with teachers’ social and emotional competence and well-being.

With reference to two measures of self-efficacy assessing work-related aspects (i.e., professional self-efficacy measuring different tasks and challenges that teachers face daily, and self-efficacy as an emotional socializer toward students’ emotions that is specifically related to beliefs about one’s own role and capabilities in the socialization of students’ emotions), we have to note that the two groups of teachers presented different mean scores at the pre-test. One of the characteristics of the present quasi-experimental approach was that the experimental group was made up of teachers who voluntarily decided to take part in the training program, while the control group was recruited from teachers of the same scholastic institutions that did not decide to participate in the training: this could suggest that teachers in the experimental group may have been motivated to participate because they were particularly eager to train on topics that they perceived as salient at that moment of their professional career, or which they felt to take care of because problematic. That said, in line with our hypotheses, teachers in the experimental group showed significant improvement in both forms of self-efficacy compared to the control group; moreover, at the post-test, the mean scores were no longer significantly different. Self-efficacy is an individual characteristic that can be trained by novel information and experiences at both intrapersonal and interpersonal levels [32,33]: the experiential nature of the ME4YOU activities invited participants to test themselves in challenging situations which concern social relationships and both their own and others’ emotional activations; moreover, the group dimension of these activities provided feedback from peers in an environment of mutual care. Finally, the trainers continuously underlined the close link between professional characteristics and personal characteristics. All the above conditions provided teachers the opportunity to gain greater awareness about their own functioning (in terms of both strengths and weaknesses) and achieve greater confidence in their own abilities as professionals. The benefits of increased levels of self-efficacy in teachers—beyond increasing their health and well-being [2]—are numerous. For instance, research has highlighted that professional self-efficacy in teachers affects students’ outcomes [38]; moreover, professional self-efficacy affects more general levels of work engagement (i.e., the degree of vigor, dedication, and absorption toward work) both directly and indirectly via reflection and resilience [39]. Further, the specific facet of self-efficacy concerning the socialization of students’ emotions is related to coaching practices involving attention and caring toward students’ emotional experiences [25]. In this regard, it would be desirable to expand and build on these promising results by providing evidence concerning the effects of the ME4YOU training program, not only on teachers’ self-efficacy but also on these more distal processes.

Promising results also emerged with reference to variables pertaining to general aspects of emotional and relational functioning, even if they were not completely in line with our hypotheses. There was evidence—which, however, requires future investigations regarding the magnitude of the effects—indicating that teachers who took part to the ME4YOU training program reduced their denial approach toward own emotional experience and increased their perception of authenticity, but levels of emotional self-efficacy toward own emotions were not impacted. The experiential activities realized during the training, thanks to the group dynamics and the mediational role of the trainer, stimulated participants to contact and deal with their own emotional world (including emotional activations of unpleasant states), to recognize their own needs, and to train their own personal relational skills with peers; however, we must underline that these activities started from stimuli that concern the workplace and the work experience, and the activation of dynamics that go beyond the school dimension could have been limited. Although research is increasingly highlighting the close link between teachers’ professional role and personal characteristics [5,9,23,24], it is not surprising that training activities such as the ME4YOU program had a stronger impact on the individual aspects of the teacher directly connected to the work experience, while the impact on their general functioning as a person was more limited, although present. We could hypothesize that the ME4YOU training program has activated a deeper reflexivity process with reference to beliefs and behaviors more closely related to the professional area, while it has activated a level of reflection—which does not necessarily result in a generalized and extended change in beliefs and behaviors—with reference to the personal area. It is important to continue testing these aspects in future research, maybe including qualitative assessment tools that allow us to access the perception of the impact of the ME4YOU on different aspects of one’s own functioning as a teacher and as a person.

Prior to concluding, we must highlight some limitations that affect the present study. First, as stated, teachers participated in the training program voluntarily, and the control group was recruited from colleagues who did not decided to participate (e.g., maybe because they were resistant to such interventions or because they did not perceived the emotional and relational facets of teaching as salient aspects in their work experience): this aspect might account for different mean scores at the pre-test in some of the measures adopted here. Moreover, the results obtained might not be generalizable to all teachers. Second, we limited the evaluation of the efficacy of the ME4YOU program at the post-test, and follow-up evaluations would have been desirable to assess its effects over time. Third, as reported above, we tested the effects of ME4YOU only on teachers (i.e., the direct beneficiaries of the intervention), and it would be interesting to test them according to the Prosocial Classroom Model by Jennings and Greenberg [3] (i.e., also on the indirect beneficiaries, such as students and colleagues); with regard to the direct beneficiaries, it would be interesting to expand the assessment to other tools and/or other variables relevant to individual professional and personal processes (e.g., to overcome some current limitations, such as the low reliability value shown by the variable denial of own emotions), also adding qualitative tools alongside the quantitative ones. A specific reflection for future research concerns the possibility of using observational tools, both to assess the evolution of interactive dynamics within the training group and to assess the impact in the classrooms, e.g., in terms of the quality of interactions between teachers and their pupils, or with reference to outcomes in the students’ social and emotional development.

## 5. Conclusions

Despite the above-reported limitations and the need for further studies to consolidate the extant evidence, this study highlights that the ME4YOU training program has an impact in supporting teachers’ socio-emotional competence and well-being. Specifically, this program seemed to attract the attention of teachers that recognized the importance of emotional and relational facets related to their profession and/or particularly needed to train these aspects. Participation in this training program seemed to promote both individual adaptive processes closely linked to teachers’ work experience and some aspects of their general functioning (although with a more limited impact and with the need to obtain in-depth evidence in this regard). These results are in line with one of the assumptions of the Prosocial Classroom Model by Jennings and Greenberg [3]: the close connection between professional role and personal characteristics. They also encourage the future testing of aspects of this model that were not directly investigated in the present study, namely whether the promotion of teachers’ well-being and social and emotional competence through the ME4YOU training program have an adaptive impact on the indirect beneficiaries (first of all, the students). Taking these considerations together, we can consider the ME4YOU program a promising training experience that can be added to the number of more consolidated programs which aim to take care of teachers’ health and socio-emotional function, representing both a caring practice on the part of a school organization that aims to promote health among its professionals and a potential investment to generate health in the whole school system.

## Figures and Tables

**Table 1:** Professional self-efficacy over time by group.

	Pre-Test	Post-Test
	M (SD)	M (SD)
Experimental group *	3.52 (0.52)	3.64 (0.46)
Control group *	3.72 (0.57)	3.64 (0.46)

Notes: * there were 4 missing values in each group.

**Table 2:** Self-efficacy as emotional socializer toward students over time by group.

	Pre-Test	Post-Test
	M (SD)	M (SD)
Experimental group	3.43 (0.48)	3.61 (0.46)
Control group	3.65 (0.55)	3.67 (0.51)

**Table 3:** Self-efficacy toward own emotions over time by group.

	Pre-Test	Post-Test
	M (SD)	M (SD)
Experimental group *	3.70 (0.46)	3.73 (0.49)
Control group	3.88 (0.49)	3.87 (0.53)

Notes: * there were 2 missing values in the experimental group.

**Table 4:** Denial of own emotions over time by group.

	Pre-Test	Post-Test
	M (SD)	M (SD)
Experimental group *	2.66 (0.64)	2.47 (0.61)
Control group *	2.54 (0.56)	2.52 (0.63)

Notes: * there were 2 missing values in each group.

**Table 5:** Authenticity over time by group.

	Pre-Test	Post-Test
	M (SD)	M (SD)
Experimental group *	4.59 (0.87)	4.73 (0.85)
Control group **	4.67 (0.81)	4.61 (0.84)

Notes: * there were 4 missing values in the experimental group; ** there were 3 missing values in the control group.

## Data Availability

There are no unpublished data available. The corresponding author can be contacted on this matter.

## References

[1] Dreer B. (2023). On the outcomes of teacher wellbeing: A systematic review of research. Front. Psychol..

[2] Hascher T., Waber J. (2021). Teacher well-being: A systematic review of the research literature from the year 2000–2019. Educ. Res. Rev..

[3] Jennings P.A., Greenberg M.T. (2009). The Prosocial Classroom: Teacher social and emotional competence in relation to student and classroom outcomes. Rev. Educ. Res..

[4] Schonert-Reichl K.A. (2017). Social and emotional learning and teachers. Future Child..

[5] Baroncelli A., Iacopino M., Facci C., Tomberli L., Ciucci E. (2024). The boundaries between personal life and professional role: A proposal to apply some principles of the Structural Family Therapy by Salvador Minuchin to teachers. J. Educ. Teach..

[6] Burić I., Slišković A., Penezić Z. (2019). Understanding teacher well-being: A cross-lagged analysis of burnout, negative student-related emotions, psychopathological symptoms, and resilience. Educ. Psychol..

[7] Montgomery C., Rupp A.A. (2005). A meta-analysis for exploring the diverse causes and effects of stress in teachers. Can. J. Educ..

[8] Ornaghi V., Conte E., Cavioni V., Farina E., Pepe A. (2019). The role of teachers’ socio-emotional competence in reducing burnout through increased work engagement. Front. Psychol..

[9] Nias J. (1996). Thinking about feeling: The emotion in teaching. Camb. J. Educ..

[10] Kariou A., Koutsimani P., Montgomery A., Lainidi O. (2021). Emotional labor and burnout among teachers: A systematic review. Int. J. Environ. Res. Public Health.

[11] Wharton A.S. (2009). The sociology of emotional labor. Annu. Rev. Sociol..

[12] Leroy H., Anseel F., Dimitrova N.G., Sels L. (2013). Mindfulness, authentic functioning, and work engagement: A growth modeling approach. J. Vocat. Behav..

[13] Van den Bosch R., Taris T.W., Schaufeli W.B., Peeters M.C.W., Reijseger G. (2019). Authenticity at work: A matter of fit?. J. Psychol..

[14] Feucht F.C., Lunn Brownlee J., Schraw G. (2017). Moving beyond reflection: Reflexivity and epistemic cognition in teaching and teacher education. Educ. Psychol..

[15] Moore A., Townsend T., Bates R. (2007). Understanding the social Self: The role and importance of reflexivity in schoolteachers’ professional learning. Handbook of Teacher Education. Globalization, Standards and Professionalism in Times of Change.

[16] Parrello S., Iorio I., De Rosa B., Sommantico M. (2020). Socio-educational work in at-risk contexts and professional reflexivity: The multi-vision group of “Maestri di Strada”. Soc. Work Educ..

[17] Ryan A., Webster R.S., Webster R., Whelen J. (2019). Teacher reflexivity: An important dimension of a teacher’s growth. Rethinking Reflection and Ethics for Teachers.

[18] Saarni C. (1999). The Development of Emotional Competence.

[19] Mahoney J.L., Weissberg R.P., Greenberg M.T., Dusenbury L., Jagers R.J., Niemi K., Schlinger M., Schlund J., Shriver T.P., VanAusdal K. (2021). Systemic social and emotional learning: Promoting educational success for all preschool to high school students. Am. Psychol..

[20] Zins J.E., Weissberg R.P., Wang M.C., Walberg H.J. (2004). Building Academic Success on Social and Emotional Learning: What Does the Research Say?.

[21] Taylor R.D., Oberle E., Durlak J.A., Weissberg R.P. (2017). Promoting positive youth development through school-based social and emotional learning interventions: A meta-analysis of follow-up effects. Child Dev..

[22] Durlak J.A., Weissberg R.P., Dymnicki A.B., Taylor R.D., Schellinger K.B. (2011). The impact of enhancing students’ social and emotional learning: A meta-analysis of school-based universal interventions. Child Dev..

[23] Cavioni V., Grazzani I., Ornaghi V., Agliati A., Gandellini S., Cefai C., Camilleri L., Bartolo P., Tatalović Vorkapić S., Golob L. (2023). A multi-component curriculum to promote teachers’ mental health: Findings from the PROMEHS program. Int. J. Emot. Educ..

[24] Grazzani I., Martinsone B., Simoes C., Cavioni V., Pepe A. (2024). Assessing teachers’ social and emotional competences: The validation of SECTRS in Italy, Latvia, and Portugal. Int. J. Emot. Educ..

[25] Ciucci E., Baroncelli A. (2024). Meta-emotion philosophy in teachers from kindergarten to middle school. Curr. Psychol..

[26] Ciucci E., Baroncelli A., Toselli M. (2015). Meta-emotion philosophy in early childhood teachers: Psychometric properties of the Crèche Educator Emotional Styles Questionnaire. Early Child. Res. Q..

[27] Gottman J.M., Katz L.F., Hooven C. (1996). Parental meta-emotion philosophy and the emotional life of families: Theoretical models and preliminary data. J. Fam. Psychol..

[28] Lagacé-Séguin D.G., Coplan R.J. (2005). Maternal emotional styles and child social adjustment: Assessment, correlates, outcomes and goodness of fit in early childhood. Soc. Dev..

[29] Lunkenheimer E.S., Shields A.M., Cortina K.S. (2007). Parental emotion coaching and dismissing in family interaction. Soc. Dev..

[30] Jennings P.A., Brown J.L., Frank J.L., Doyle S., Oh Y., Davis R., Rasheed D., DeWeese A., DeMauro A.A., Cham H. (2017). Impacts of the CARE for Teachers Program on teachers’ social and emotional competence and classroom interactions. J. Educ. Psychol..

[31] Chesak S.S., Khalsa T.K., Bhagra A., Jenkins S.M., Bauer B.A., Sood A. (2019). Stress Management and Resiliency Training for public school teachers and staff: A novel intervention to enhance resilience and positively impact student interactions. Complement. Ther. Clin. Pract..

[32] Bandura A. (1997). Self-Efficacy: The Exercise of Control.

[33] Bandura A. (2006). Toward a psychology of human agency. Perspect. Psychol. Sci..

[34] Caprara G.V., Barbaranelli C., Steca P., Malone P.S. (2006). Teachers’ self-efficacy beliefs as determinants of job satisfaction and students’ academic achievement: A study at the school level. J. Sch. Psychol..

[35] Caprara G.V. (2001). La Valutazione Dell’Autoefficacia. Costrutti e Strumenti.

[36] Di Fabio A. (2014). Authenticity Scale: A first contribution to validation of the Italian version. Couns. G. Ital. Ric. Appl..

[37] Wood A.M., Linley P.A., Maltby J., Baliousis M., Joseph S. (2008). The authentic personality: A theoretical and empirical conceptualization and the development of the Authenticity Scale. J. Couns. Psychol..

[38] Klassen R.M., Tze V.M.C. (2014). Teachers’ self-efficacy, personality, and teaching effectiveness: A meta-analysis. Educ. Res. Rev..

[39] Heng Q., Chu L. (2023). Self-efficacy, reflection, and resilience as predictors of work engagement among English teachers. Front. Psychol..

